# Ca_3_Co_4_O_9_-Based Planar Thermoelectric Gas Sensor with High Sensitivity Fabricated by Powder Aerosol Deposition for Application in Harsh Environments

**DOI:** 10.3390/s26154864

**Published:** 2026-08-02

**Authors:** Benedikt Streibl, Thomas Wöhrl, Daniel Paulus, Jaroslaw Kita, Daniela Schönauer-Kamin, Gunter Hagen, Ralf Moos

**Affiliations:** Department of Functional Materials, Zentrum für Energietechnik (ZET), University of Bayreuth, D-95440 Bayreuth, Germany

**Keywords:** thermoelectric gas sensor, calcium cobaltite, powder aerosol deposition, flue gas

## Abstract

Increasingly stringent emission regulations in combustion systems drive the demand for robust, cost-effective gas sensors capable of operating under harsh, high-temperature conditions. Thermoelectric ceramic gas sensors represent a promising approach. Their application, however, may be limited owing to a low response. In this work, calcium cobaltite (CCO), a p-type thermoelectric oxide with a Seebeck coefficient higher than most metals used for thermocouples, is investigated as an alternative material to enhance sensor performance. CCO powder was synthesized via the mixed oxide route and deposited as dense ceramic films onto alumina substrates at room temperature using the powder aerosol deposition method (PAD). The thermoelectric properties of the deposited films were characterized up to 850 °C, with the Seebeck coefficient showing only minor dependencies on variations in oxygen and water vapor concentrations. Following these results, planar exothermic gas sensors based on Au/CCO thermocouples utilizing laser-cut polyimide masks for patterning the PAD films were fabricated and compared to reference sensors with screen-printed metallic Au/Pt thermocouples. Laboratory gas measurements with CO and hydrocarbons demonstrated that the Au/CCO-based sensors exhibited an average sensitivity increase by a factor of 8–9, while maintaining high linearity and low cross-sensitivity to variations in oxygen and humidity. Furthermore, the additive response to gas mixtures was confirmed. Initial tests in real flue gas from a wood-burning stove showed an excellent correlation between the sensor signal and relevant flue gas components (measured using precise gas analyzers). The presented results highlight the potential of calcium cobaltite as a thermoelectric material for high-temperature gas sensors based on the exothermic principle and demonstrate the suitability of the PAD method for fabricating fine-structured functional films.

## 1. Introduction

### 1.1. Motivation

Protection of the environment and of human health has become a key issue in all areas of industry over the past decades, as the impact of pollutants on the environment and on life cannot be underestimated [1,2,3,4]. Regulatory frameworks aim to limit emissions from combustion processes (e.g., the recently implemented Euro 7 standard that sets stringent limits on various emissions from motor vehicles [5]). Increasingly strict regulations apply to almost any area of industry in order to comply with certain emissions limits across the globe [6,7,8].

Even wood log burning in private households is now subject to emission regulations [9]. This is driving up demand for cost-effective sensor technology to measure the concentration of flue gases like volatile organic components (VOC), hydrocarbons (HCs) or carbon monoxide (CO). They are intended to support control of the combustion process. Since conditions in flue gases are often harsh, a robust sensor principle is necessary [10]. Planar sensors (mostly based on ceramic technology) have been proven to be a potential option [11,12,13], as they can be operated at high temperatures, while the sensor setup remains simple.

For instance, Zhang et al. showed that emissions from a commercial fireplace can be reduced by up to 90% by utilizing an HC sensor (measuring total HCs and CO) together with the oxygen concentration, flue gas temperature, and air mass flow [14].

Ohja et al. showed that using a similar setup for an automatic air control system (HC sensor, flue gas temperature and oxygen concentration) can reduce the emissions of CO and unburned hydrocarbons by up to 80%. Both mixed-potential sensors and metal oxide sensors could be implemented with varying degrees of success [15].

Several approaches for reliable gas sensors in harsh conditions use thermoelectric catalytic gas sensors. Such sensors typically comprise a catalytically active region and an inert reference region, both thermally coupled to a thermopile or thin- or thick-film thermoelectric structure [16,17,18,19,20,21].

When combustible gas species, such as HCs or CO, are present in the surrounding atmosphere, they undergo heterogeneous oxidation at the catalytic surface. This reaction is exothermic and leads to a localized temperature increase in the active region relative to the reference. The resulting temperature gradient across the thermoelectric element induces a voltage proportional to the temperature difference, according to the Seebeck coefficient of the material system.

Under steady-state conditions, the magnitude of the generated thermoelectric voltage reflects the balance between the heat released by the catalytic reaction and the heat dissipated into the environment. Consequently, the sensor signal can be related to the concentration and reactivity of the target gas components. Due to its calorimetric operating principle, the thermoelectric gas sensor inherently measures the total exothermic activity of reactive gas mixtures rather than selectively detecting individual species.

This makes such sensors particularly suitable for applications in combustion monitoring and control, where the overall concentration of unburned or partially oxidized species is of primary interest. However, the sensor response can be influenced by heat losses and ambient temperature fluctuations; the inherently non-selective calorimetric principle makes it difficult to distinguish between different combustible gas species. The main disadvantage is their relatively low signal amplitude, since the temperature differences generated by the catalytic reaction are typically small and commonly used thermoelectric materials exhibit comparatively low Seebeck coefficients. This results in low sensor signals, which makes precise measurement devices mandatory.

This limitation motivates the present study, which aims to enhance the sensitivity of an existing thermoelectric gas sensor, by applying an oxide thermoelectric material (calcium cobaltite Ca_3_Co_4_O_9_, CCO) to replace the platinum leg. Since CCO cannot withstand typical ceramic processing temperatures (>926 °C [22]), it should be processed using the novel room temperature powder aerosol deposition method instead of screen-printing. As the CCO films require fine patterning, a laser-cut polyimide mask is utilized. Sensitivities and cross-sensitivities of the sensors are investigated and an exemplary initial test of real flue gas in a wood-burning stove is shown.

### 1.2. Catalytic Thermoelectric Gas Sensors: Previous Work and Basic Setup

**Figure 1:**
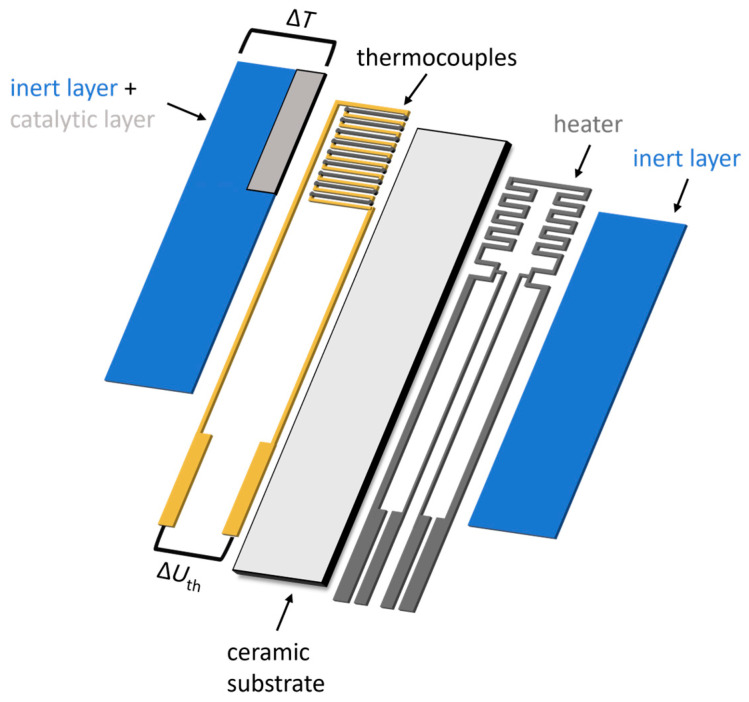
Exploded view of the planar sensor substrates, illustrating the individual layers. The thermocouples are made of two distinct materials.

The sensor principle used in this study is based on previous work [23,24]. The planar high-temperature HC sensors are based on aluminum oxide substrates. Functional films are deposited by screen-printing, as illustrated in Figure 1. A platinum heater meander with supply lines is first applied to the backside. Its resistance is monitored via four-wire measurement and used to control the substrate temperature through Joule heating along a predefined temperature curve [25]. The heater is then covered with an inert film. On the front side, one or more thermocouples made of materials A and B are deposited and connected in series. These are positioned at the sensor tip, corresponding to the heater location on the opposite side. The thermocouples are covered by an inert film on one side and a catalytic active film on the other. Typical operation temperatures of the sensor tip are between 300 °C and 600 °C, since such temperatures are needed to oxidize the reducing components, CO or HCs [26].

If reactive gases are present, they undergo exothermic oxidation at the catalyst, leading to a local temperature increase. Since the catalyst is applied only to one side, a temperature difference develops across the thermocouples. This gradient generates a thermoelectric voltage *U*_th_ across *n* thermocouples [27], according to the following equation:

*U*_th_ = *n*·(*S*_A_ − *S*_B_)·(*T*_cat,hot_ − *T*_inert,cold_) = *n*·*S*_A/B_·∆*T* (1)

where the effective Seebeck coefficient *S*_A/B_ of the materials A and B is used (*S*_A/B_ is independent of the layer thicknesses of materials A and B).

Because the temperature difference is only a few degrees [26] and most Seebeck coefficients are in the µV/K range, the resulting thermoelectric is generally limited to the µV range. For instance, the widely used Au–Pt combination exhibits a temperature-dependent Seebeck coefficient of 6 to 20.5 µV/K between 0 and 1000 °C [28]. Substituting one of these materials with a higher-Seebeck material can significantly enhance the sensor signal. In this work, calcium cobaltite Ca_3_Co_4_O_9_ (CCO) is used. It exhibits a temperature-dependent Seebeck coefficient of 120 to 210 µV/K between 0 to 800 °C [29,30].

## 2. Materials and Methods

### 2.1. Synthesis of Calcium Cobaltite and Powder Aerosol Deposition

Calcium cobaltite is a p-type thermoelectric oxide with a layered misfit structure, consisting of a triple rock salt-type Ca_2_CoO_2_ sublattice and a CdI_2_-type CoO_2_ sublattice [31]. The CCO powder used in this work was manufactured via the mixed oxide route, as described in [32]. A stoichiometric mixture of CaCO_3_ (99% low-alkali, Riedel-de Haën, Seelze, Germany) and Co_3_O_4_ (99.97%, chemPUR, Karlsruhe, Germany) was wet-milled with zirconia balls in cyclohexane (99.5%, Carl Roth, Karlsruhe, Germany) using a planetary mill (Pulverisette 5, Fritsch, Idar-Oberstein, Germany) followed by separation, cleaning, and drying of the powder. The dried powder was then calcined at 900 °C for 12 h and subsequently reground using the same milling procedure to obtain the final powder. Further details are reported in [32].

The CCO thermoelectric leg was fabricated using the powder aerosol deposition (PAD) method, as it has been shown to provide improved thermoelectric properties compared to other fabrication techniques [33]. Additional information about the microstructure of the deposited CCO films can also be found in [33]. PAD also enables the deposition of dense films on planar HC sensor substrates, although additional fine structuring of these films is required, as discussed in the following section. Extensive fundamentals about the PAD method are provided in the literature. The PAD method makes it possible to produce dense ceramic films directly from a powder, even at room temperature [34,35,36,37,38,39].

For PAD, an aerosol stream is generated by fluidizing a powder bed with a continuous flow of a carrier gas (in this case oxygen). This stream is transported into a vacuum deposition chamber, where particles are accelerated through a nozzle and impact the substrate (see Supplementary Information (SI), Figure S1). Successful deposition requires optimal kinetic energy, depending on factors such as particle size and morphology [40,41]. The film formation occurs in two stages, anchor-layer formation and subsequent growth. Since the resulting films exhibit compressive stress, thermal post-treatment is often required [42,43,44]. For CCO, this treatment can further enhance the Seebeck coefficient [33]. This was achieved here during the firing of the screen-printed films and the initial sensor operation. PAD films show high adhesion to the substrate if the hardness of the powder and substrate are similar, which makes it an excellent choice for applying ceramic films on ceramic substrates [35,39].

### 2.2. Sensor Manufacturing

**Figure 2:**
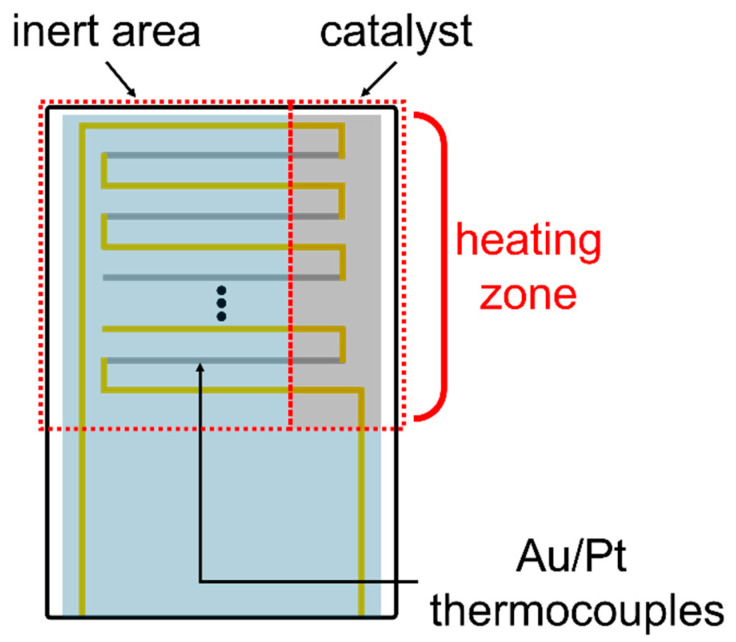
A typical sensor tip from previous work, as it is used in this study. The Au/Pt thermocouples are connected in series and are partially covered by an inert film and a catalyst film. The area of the platinum heater on the back side of the sensor is denoted as the “heating zone”.

A typical tip of a sensor from previous work [23,24,26] is shown in Figure 2. It has also been used in this study and serves as the reference.

A ceramic planar substrate serves as an electrically insulating support. All functional elements were fabricated by screen-printing: the thermocouple structures on the front side consist of platinum and gold, while the heater on the backside is, likewise, made of platinum. The heater, the conductive tracks, and the inert regions were covered with a glass–ceramic insulating film. The aim of this study is to replace the platinum leg of the thermocouple with a CCO leg manufactured by the PAD.

The following section describes the production of the sensor films. On the back side, the heating element was first deposited by screen-printing using a platinum paste (9141R, DuPont, Neu-Isenburg, Germany), followed by drying at 110 °C, and subsequent firing. A glass–ceramic cover film (QM42, DuPont, Neu-Isenburg, Germany) was then printed on top, leaving only the contact pads exposed, and likewise dried and fired. The corresponding firing profiles are provided in Supplementary Information (SI), Tables S1–S3.

**Figure 3:**
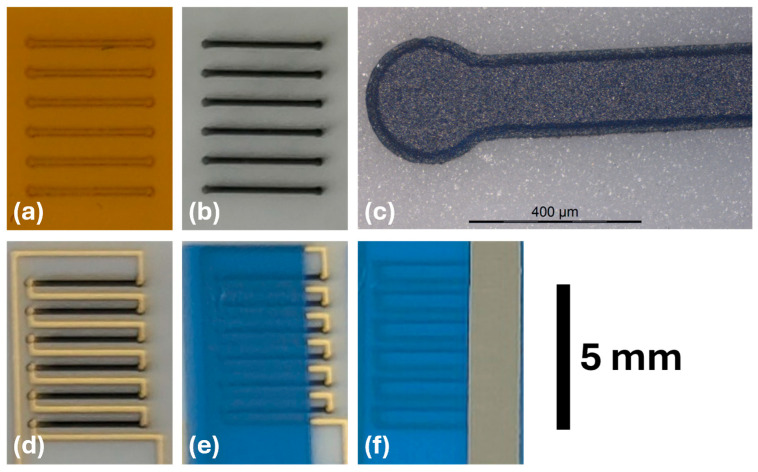
Different steps for producing the sensors, showing one sensor tip of the substrate: (**a**) laser-cut polyimide tape, with the cut-out parts being removable; (**b**) CCO sprayed with the PAD method, polyimide mask already removed; (**c**) microscope image of one CCO line showing a darker outline due to grooves made by the laser; (**d**) gold lines printed on top; (**e**) inert cover film printed on top, leaving room for the catalyst film in the top right corner of the sensor; and (**f**) catalyst film printed on top.

The following further describes the fabrication of the front-side films. For Au/CCO sensors, a structured calcium cobaltite (CCO) film was first deposited by the PAD method. As patterning is required, a masking approach was employed. Due to the fine thermocouple line width (~200 µm), conventional masking tapes are unsuitable. Therefore, polyimide tape was used and subsequently patterned by laser processing (Figure 3a). This enables the selective removal of defined regions, with preliminary tests confirming minimal residue after mask removal.

The resulting CCO films exhibit a thickness of approximately 10 µm, which is higher than that of the transducer films due to the increased number of passes (see Section 2.3), yet still lower than values reported in other studies [33]. Using polyimide tape, finely structured CCO films can be fabricated by PAD in a manner comparable to unstructured films, with film thickness readily adjusted via the number of deposition passes. PAD-deposited patterned films can be seen in Figure 3b, with a microscope picture from the part of a single line in Figure 3c. After the spraying step, the gold lines are printed on top with the 5744R gold paste (DuPont, Neu-Isenburg, Germany), as shown in Figure 3d, and fired after a short drying step of about 15 min at 110 °C.

For comparison, in the case of the Au/Pt sensors, the gold electrodes were first screen-printed onto the front side with the same gold paste used for the Au/CCO sensors and then fired after a drying step at 110 °C. Afterwards, the platinum electrodes were screen-printed using the 9141R paste and fired.

After the Au/Pt and Au/CCO thermocouples have been applied to the front of the sensor substrates, an inert cover film was also applied by screen-printing (Figure 3e). The connection pads and the active area both remain free. In the final step, the catalyst film was printed onto the active area and fired (Figure 3f). This means that the fronts of the sensors were also completely covered except for the connection pads. The applied catalyst paste was prepared in-house following the procedure described in [26]. It consists of 1 wt % Pt + 1 wt % Pd in Al_2_O_3_ (71% solid content and 29% organic binder), which was proven to be a catalyst with high long-term stability under high oxygen concentrations [45,46] As a note, the CCO films experience a maximum temperature of 850 °C during the firing processes. This also marks the maximum temperatures of the Seebeck transducers, which will be the topic of the next chapter.

**Figure 4:**
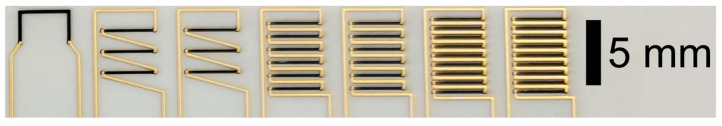
Top part of one sensor substrate with CCO and gold lines already on the substrate. One substrate consists of one sensor with one thermocouple, and two sensors with thermopiles with three, six, and nine thermocouples, respectively.

A section in the upper area of an Au/CCO sensor substrate without cover films is shown in Figure 4. As can be seen, the manufactured sensors comprise either one, three, six, or nine thermocouples. For later measurements, only one sensor with each respective thermocouple number is tested.

Unlike in previous works [23,24,26], no low-temperature co-fired ceramic (LTCC) film was applied between the Al_2_O_3_ substrate and the functional films, and no LTCC substrate was used. Due to its lower thermal conductivity [47], such a film would have the potential to increase the generated thermoelectric voltage; however, due to the unknown interactions between the glass constituents of the LTCC with the CCO at a high operation temperature, this option was not considered in this work.

### 2.3. Characterization of CCO

Since the Seebeck effect is decisive for this sensor principle, another planar sensor with a different design is used for the subsequent analysis of the Seebeck coefficient of the deposited CCO films. This sensor setup will be referred to as “Seebeck coefficient measurement transducer”, abbreviated as SCMT. The setup and explanation, together with a schematic, can be found in Supplementary Information (SI) under “Seebeck coefficient measurement transducer (SCMT)” and in Figure S2. More information on the SCMT can be found in [33,48].

To measure the Seebeck coefficient *S* of CCO at different temperatures and ambient conditions, CCO was deposited on the SCMT, with the number of passes reduced by half compared to the sensor fabrication. Therefore, the resulting film thickness was only approximately 1.5–3 µm (measured using LSM 900, Zeiss, Oberkochen, Germany). Since the Seebeck coefficient is independent of the film thickness, this does not affect its evaluation.

The Seebeck coefficient was determined by using the SCMT between room temperature and 850 °C in increments of 100 °C, following the measurement and evaluation procedure described in SI (“Seebeck coefficient measurement transducer (SCMT)”). Each temperature step was held for 2 h (3 h at 600 °C, corresponding to the typical sensor operating temperature), with heating and cooling phases of 30 min. The maximum temperature of 850 °C reflects the thermal exposure during sensor firing.

**Figure 5:**
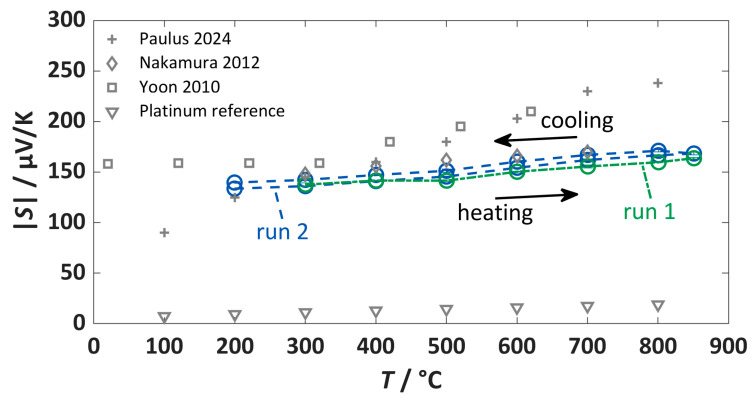
The Seebeck coefficient *S* measured during heating and cooling of two measurement runs. Points indicate the average of *S* during each temperature step; lines connecting the points serve as a guide for the eye. Additionally, the standard deviation from the average is shown as error bars for each point. The first run also marks the first time that the CCO film is being heated up and thus thermally annealed. Additionally, the values of PAD–CCO from other works [33,43,49] are shown. The absolute value of *S* from platinum is also plotted as a reference [28].

The separate heating element in front of the SCMT generated a temperature difference of approximately 5 °C across the SCMT. This modulation heater was periodically activated every 16 min for 8 min, which heats up the surrounding area. During the active phase, an applied heating voltage caused a temperature oscillation at a frequency of 12.5 mHz. The resulting thermoelectric voltage was recorded and converted into the Seebeck coefficient *S* utilizing the measured temperature difference by calculating the slope of the thermo-electric voltage over the temperature difference for each oscillation. The first measurements were conducted under a controlled gas atmosphere of 20% O_2_ in N_2_. The resulting Seebeck coefficient is shown in Figure 5 for two measurements of a single SCMT. Measurements from other studies are also shown for comparison. Additionally, the absolute value of *S* from platinum is plotted as a reference.

As can be seen in Figure 5, *S* increases slightly during the cooling process compared to the heating process. At a temperature of 600 °C, the Seebeck coefficient is approximately 153 to 167 µV/K (compared to *S*_Au/Pt_ = 19.2 µV/K at 600 °C). These results appear somewhat imprecise. However, it should be noted that despite the simplicity of the measurement setup, accurately measuring the Seebeck coefficient is challenging. This was highlighted in a recent round-robin study on the transport properties of a standard bismuth telluride, which reported discrepancies in the determined Seebeck coefficient in the range of ±5% [50]. Nevertheless, the results are reproducible, as a test with another SCMT with a different CCO film width showed no significant differences (Supplementary Information (SI), Figure S3).

These measurements showed that the Seebeck coefficient of CCO at 600 °C averages out to ca. 160 µV/K. Compared to results from other studies, the determined Seebeck coefficients are in a similar range, such as that observed in [43], but slightly below the results seen in [33,49].

In a separate measurement, the SCMT was subjected to temperatures of 300 °C, 600 °C, and 650 °C for 120 min each. After three of these temperature cycles, the oxygen concentration was reduced in steps of 5% from 20% to 5%, while the total flow rate was kept at 200 mL/min by correspondingly increasing the nitrogen fraction from 80% to 95%. A detailed investigation of the dependence of the Seebeck coefficient of CCO on the oxygen concentration is required, as it is well established that the Seebeck coefficient of oxide materials is strongly governed by defect chemistry and the associated oxygen stoichiometry [51].

This procedure mimics typical HC sensor operating conditions, with a nominal temperature of 600 °C and oxygen concentrations between 5% and 20%. In combination with the additional temperature levels, the measurement allows for an assessment of repeatability during thermal cycling of the CCO films and, consequently, of the sensor performance.

**Figure 6:**
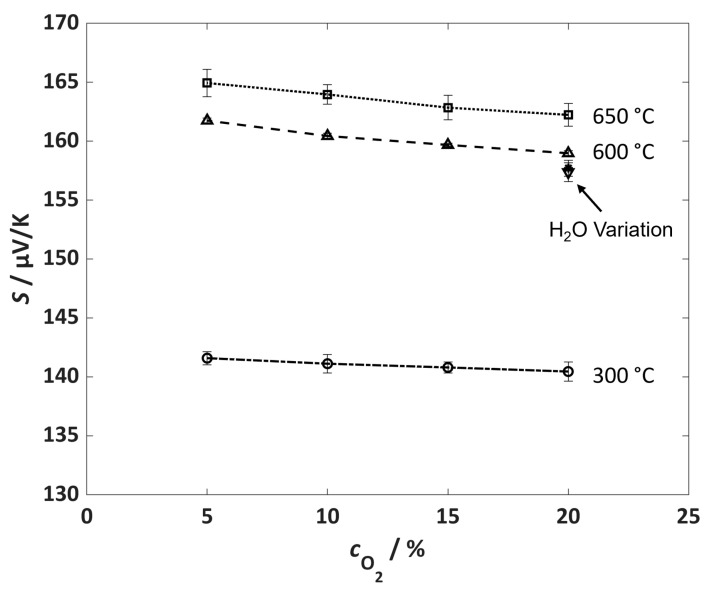
Seebeck coefficient of powder aerosol deposited CCO as a function of the oxygen concentration in N_2_ at different temperatures. For each oxygen concentration, three temperature cycles were performed. The resulting coefficients were averaged (symbols), with standard deviations indicated by error bars. Additionally, results from a separate measurement at 600 °C, with and without the addition of 2.3% water (at 20% O_2_ in N_2_) are shown for comparison.

Figure 6 shows that, at 300 °C, the Seebeck coefficient remains essentially unaffected by variations in oxygen concentration, with the scatter across the three cycles at each concentration being comparable to the variation between different concentrations. At higher temperatures, only a very slight increase in *S* is observed with decreasing oxygen concentrations, from 160 µV/K to 162 µV/K at 600 °C and from 163 µV/K to 165 µV/K at 650 °C.

In a separate experiment, the influence of water vapor on *S* was examined. As shown in Figure 6, the addition of 2.3% H_2_O does not lead to a measurable change in the Seebeck coefficient. Instead, *S* fluctuates around a mean value of approximately 158 µV/K, with a maximum deviation of about 2 µV/K, showing no systematic change even after prolonged exposure. For the subsequent sensor application, this indicates that variations in the sensor signal are not caused by the interactions of oxygen or water with the CCO film.

### 2.4. Sensor Test Setup

#### 2.4.1. Gas Sensor Test Bench in the Lab

The manufactured sensors were evaluated for their sensitivity to individual gases using a dedicated gas sensor test system. The setup consists of multiple mass flow controllers (MFCs) for controlled dosing of gas mixtures into a heated measuring chamber (see Supplementary Information (SI), Figure S4), in which the sensors are installed. During all measurements, a constant total flow rate of 6 L/min was maintained. The sensor voltage signals were recorded with a Keithley 2700 multimeter (Tektronix, Beaverton, OR, USA). The oxygen concentration of the gas mixture was monitored upstream of the chamber using a Bosch LSU 5.2 wideband lambda sensor (Bosch, Gerlingen-Schillerhöhe, Germany) in combination with an ETAS lambda measurement module. Downstream, the exhaust gas was analyzed with an FTIR system (MultiGas 2030, MKS Instruments, Munich, Germany) to determine actual gas concentrations. This is necessary because deviations between the set and delivered concentrations can occur; however, these deviations were found to be consistent across all experiments.

#### 2.4.2. Application in Flue Gas (Wood-Burning Stove)

For investigations under real flue gas applications, a commercially available wood-burning stove (type UNICA from LEDA) was used, with dried spruce wood as fuel. The stove pipe was modified by adding several threaded connectors, allowing for the integration of sensors and measurement devices. The exhaust pipe was connected to a 17 m tall chimney operating under natural draft, i.e., without controlled air supply. A photograph of the experimental setup is provided in Supplementary Information (SI), Figure S5.

At the measurement location, the sensor and a lambda probe were installed. Slightly above this position, a connection for a flame ionization detector (FID, Emerson, St. Louis, MO, USA) was placed to measure the total hydrocarbon concentration. In addition, the same device integrates two non-dispersive infrared (NDIR) detectors to determine CO and CO_2_ concentrations.

Compared to an FTIR system, an FID is less susceptible to contamination and easier to maintain. The sensor and the FID sampling points were positioned with minimal spatial offset to ensure comparable gas compositions at both measurement locations.

## 3. Results

### 3.1. Sensor Characterization

**Figure 7:**
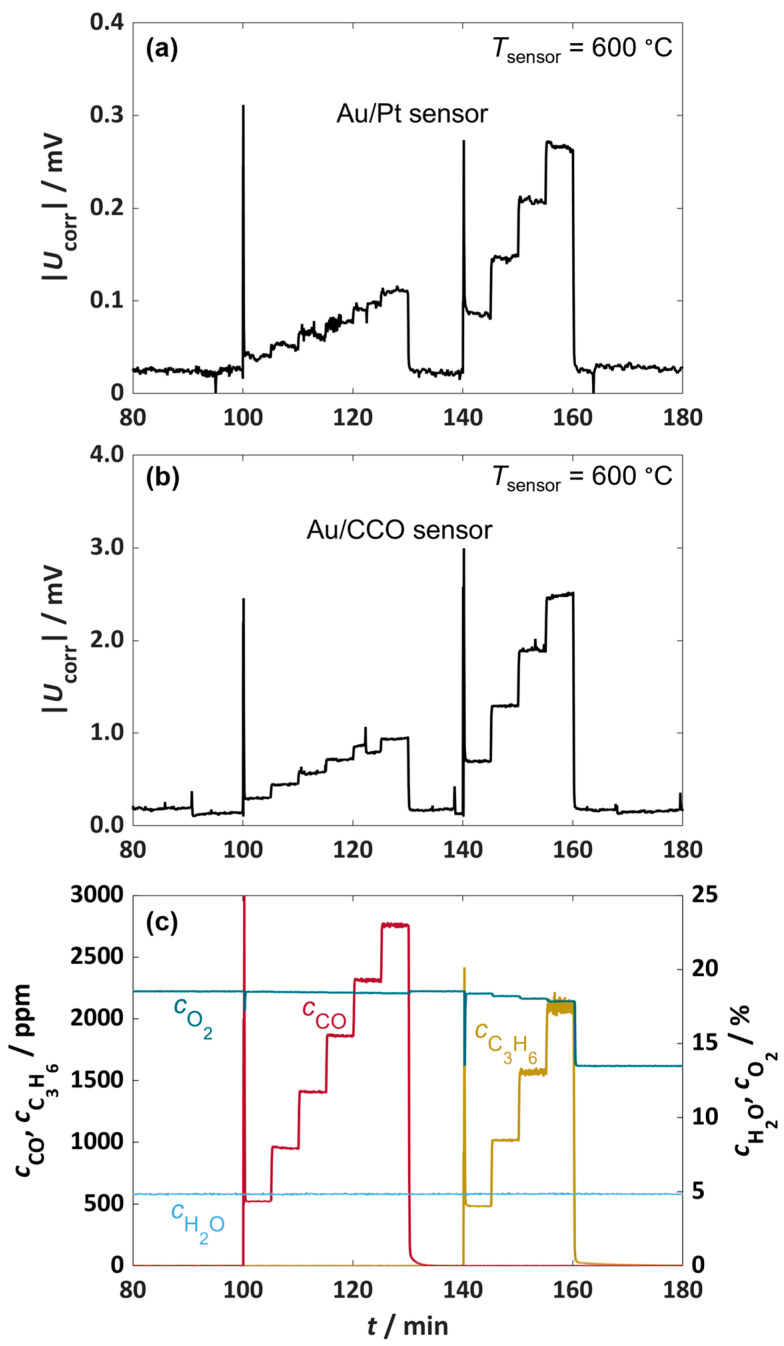
One measurement cycle showing: (**a**) sensor signal of the Au/Pt sensor; (**b**) sensor signal of the Au/CCO sensor; and (**c**) the added gases (base gas consisted of 3% CO_2_ in N_2_; the N_2_ concentration was changed with each step and in each cycle to maintain a total flow rate of 6 L/min). After each cycle, the concentration of either O_2_ or H_2_O is changed (in this case O_2_ at *t* = 160 min). The sensor temperatures were set to 600 °C. Please note the different *y*-axis scaling in (**a**,**b**). The absolute value of the voltages is shown to more easily compare the different signs.

This section reports and discusses the sensor measurement results. All sensors were subjected to a defined measurement protocol, with the operating temperature being set to 600 °C using the back side heater. An excerpt of raw data from sensors, comprising nine thermocouples, is shown in Figure 7. Since the sensors exhibit a non-zero baseline voltage even in the absence of test gases, the signals were offset-corrected and are, therefore, reported as *U*_corr_. This effect is well-known and originates from a residual temperature difference over the thermocouples (Δ*T* = *T*_cat,hot_ − *T*_inert,cold_). The temperature gradients may result from the installation position of the sensor, asymmetry of the sensor design, or variances in the manufacturing of the catalyst film (e.g., inhomogeneous film thickness), which results in an inhomogeneous temperature distribution [24,52].

For better visual comparison, the absolute values |*U*_corr_| are shown for all sensors. Because the combined Seebeck coefficient of Au/CCO is negative and that of Au/Pt is positive (at 600 °C: *S*_Au_ ≈ +3.7 µV/K and *S*_Pt_ ≈ −15.5 µV/K [28]), while *S*_CCO_ ≈ +165 µV/K, this results in *S*_Au/Pt_ ≈ 19.2 µV/K and *S*_Au/CCO_ ≈ −161.3 µV/K), which causes the signal of the Au/CCO sensors to move in the opposite direction compared to the signal of the Au/Pt sensors.

The test gases CO and C_3_H_6_ were supplied individually and concentrations were increased by 500 ppm every five minutes up to a defined maximum (3000 ppm for CO, 2000 ppm for C_3_H_6_), followed by flushing with a base gas mixture constituting one measurement cycle. These cycles were repeated for different O_2_ and H_2_O concentrations, as shown in Figure 7 (e.g., at *t* = 160 min, where the oxygen concentration decreases from 20% to 15%). Both test gases were never supplied simultaneously, but always separately. Figure 7 shows that both sensor types exhibit a similar qualitative response, with signals increasing with the rising gas concentration. It is essential to note that the axes of the sensor signals are scaled differently. Notably, the Au/CCO sensor produces a significantly higher response; for example, at 1000 ppm CO, it yields approximately 1 mV compared to 0.13 mV for the Au/Pt sensor. The noise behavior of the Au/CCO sensor is lower than that of the Au/Pt sensor, but the noise behavior of the remaining Au/Pt sensors is generally lower than the sensor shown in Figure 7.

For further evaluation, the averaged sensor signal is plotted against the gas concentration.

**Figure 8:**
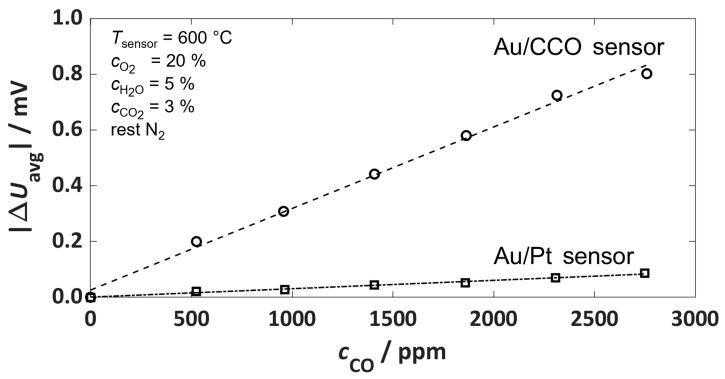
Average voltages |∆*U*_avg_| per CO concentration plateau for both sensors shown in Figure 7, measured at sensor temperatures of 600 °C. Symbols represent the average voltage during each step/plateau, the dotted lines represent a linear interpolation between the points.

Figure 8 shows the average voltages |∆*U*_avg_| of the Au/Pt sensor and Au/CCO sensor, each with nine thermocouples, measured during a stepwise increase in CO at 20% O_2_ and 5% H_2_O (the same measurement cycle shown in Figure 7). As is apparent in Figure 8, the Au/CCO sensor can generate a significantly higher sensor signal than the Au/Pt sensor, which is already evident from the raw data. The voltage is in the range of up to approximately 0.802 mV at 2750 ppm CO, which is a significant increase compared to the Au/Pt sensor, the voltage of which rises only to approx. 0.083 mV.

**Figure 9:**
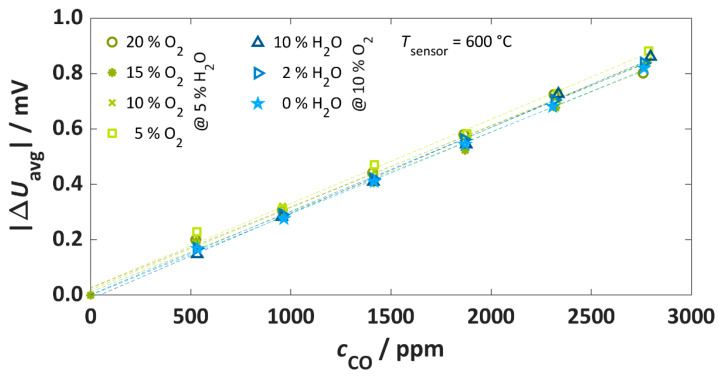
Average voltages |∆*U*_avg_| per CO concentration step for each measurement cycle at different O_2_ or H_2_O concentrations for the Au/CCO sensor shown in Figure 7 and Figure 8. Symbols indicate the average value per step/plateau (green during O_2_ variation, blue during H_2_O variation), the dotted lines represent linear interpolations for the voltages during one measurement cycle, i.e., at a constant O_2_ and H_2_O concentration.

Figure 9 shows the averaged voltages |∆*U*_avg_| of the Au/CCO sensor with nine thermocouples, which were generated during the stepwise increase in CO, with variations in the oxygen and water concentrations (the diagram with C_3_H_6_ instead of CO can be seen in Supplementary Information (SI), Figure S6). The difference between the individual variations is small and the increase of the sensor signal is almost independent of the water concentration or of the oxygen concentration in the gas. This behavior was reproduced for both CO, and, to a limited extent, propane, with this sensor, and also with the other sensors, which will be discussed further below.

For better comparison, the slopes of the linear interpolations of the measured values are utilized by relating them to a concentration of 1000 ppm of the respective test gas and calculating an average value, resulting in one average sensitivity *m*^a^_x_ towards the test gas x (either CO or C_3_H_6_) per sensor.

For better visualization and comparison, the absolute value of the sensitivity was chosen. The values are shown in Table 1 for all sensors.

The sensitivities of the Au/CCO sensors for CO have increased by a factor of 8.75 on average compared to the Au/Pt sensors, while this factor is 8.92 on average for C_3_H_6_. This roughly corresponds to the ratio of the relative Seebeck coefficients (absolute values) of the thermocouples used, since at 600 °C the coefficients are *S*_Au/Pt_ = 19.2 µV/K and *S*_Au/CCO_ ≈ −161 µV/K, which is a factor of roughly 8.4. As the sensor voltage depends on the number of thermocouples, the temperature difference and the effective Seebeck coefficient (see (1)), and neither do the number of thermocouples change nor are the temperature differences significantly dissimilar, the sensitivity almost solely depends on the effective Seebeck coefficient. This also indicates that using CCO instead of Pt does not influence the temperature difference itself across the sensor substrate.

The sensitivities of the Au/CCO sensors show a maximum variance of 8.2% (CO, sensor with one thermocouple) when oxygen or water is varied, with the variance decreasing as the number of thermocouples increases (e.g., the Au/CCO sensor with nine thermocouples has a variance of 2.5% for CO, while the sensor with one thermocouple has a variance of 8.2%). Percentagewise, the variance of the Au/Pt sensors is only slightly smaller than that of the Au/CCO sensors. All sensors show a high linearity of the sensor signal as a function of the test gas concentration and exhibit behavior that is virtually independent of oxygen or water. This is further shown in the measurements of the Seebeck coefficient, where oxygen and water variations also showed no significant effect on the value of *S*.

### 3.2. Gas Mixtures

**Figure 10:**
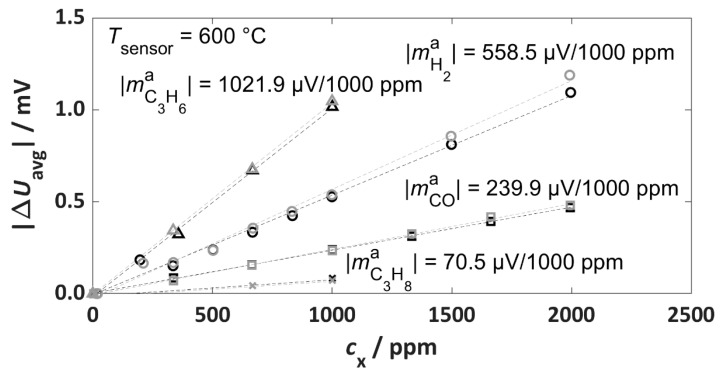
Au/CCO (nine thermocouples) sensor sensitivities for each individual test gas that was used in the gas mixture measurement at a sensor temperature of 600 °C. The sensitivities were averaged over both cycles at different atmospheric conditions; the gray symbols are for data during 5% O_2_ and 5% H_2_O and the black symbols are for data during 15% O_2_ and 3% H_2_O. The dotted lines represent linear interpolations of the points.

In a separate experiment, the Au/CCO sensor with nine thermocouples was examined in greater detail. First, four gases (CO, C_3_H_6_, C_3_H_8_ and H_2_) were admixed individually. The resulting average sensor voltages are plotted as a function of the corresponding gas concentrations to determine the sensitivities, following the same evaluation procedure used in the previous measurements (see Figure 10).

In the next step, the sensor signals were converted from a voltage into an equivalent value for CO (COe) through dividing the sensor signal by the sensitivity towards CO (see (2)), leading to the COe_Sen_ curve. A COe sum curve was derived from the admixed gas concentrations by multiplying each concentration with a weighting factor and subsequently summing the contributions (see (3)), resulting in the COe_the_ curve. The weighting factors correspond to the quotients of each sensitivity relative to the CO sensitivity, which were all taken from Figure 10:

COe_Sen_ = *U*_corr_·*m*^a^_CO_ (2)

COe_the_ = *c*_CO_ + 1/*m*^a^_CO_·(*m*^a^_C3H8_·*c*_C3H8_ + *m*^a^_C3H6_·*c*_C3H6_ + *m*^a^_H2_·*c*_H2_) (3)

When considering the sensitivity ratios in relation to CO (i.e., the weighting factors), there may be deviations from previous results with similar setups such as that used in [24], as the behavior of the catalyst and, thus, the reaction of the gases can be influenced by variables such as sensor temperature, exhaust gas temperature, diffusion through the protective cap (and thus the porosity of the protective cap), LTCC film, etc.

After the individual admixing steps, the H_2_ concentration was varied while CO was additionally admixed at two fixed concentration levels. In a final step, CO and C_3_H_6_ were applied simultaneously, with H_2_ introduced after a short delay. These measurements were conducted at 15% O_2_ and 3% H_2_O (first cycle), as well as at 5% O_2_ and 5% H_2_O (second cycle). Consistent with earlier results, the sensor exhibits a higher sensitivity to propene than to CO. In addition, the sensor is highly sensitive to H_2_, which can be attributed to the strong exothermic reaction of hydrogen [53]. Since propane (C_3_H_8_) begins to react at approx. 550–600 °C [54], only a very low response is noticeable here.

**Figure 11:**
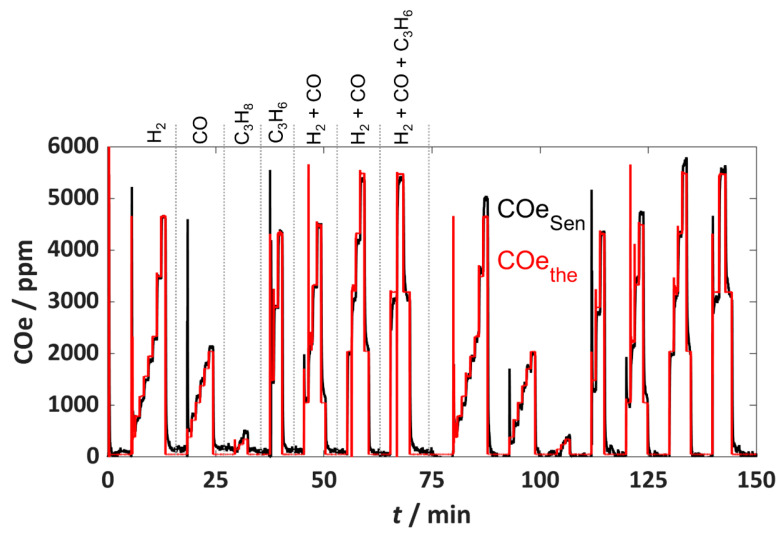
CO equivalent calculated from gas data (COe_the_) in red utilizing Equation (3) and COe_Sen_ calculated from the sensor signal calculated in black utilizing Equation (2). The dosed gases are shown above the graph during the first cycle (5% O_2_ and 5% H_2_O).

Figure 11 shows the two COe curves for the measurement. In addition, the admixed gas concentrations are indicated (only for the first 75 min). The theoretical curve COe_the_ corresponds mostly very well with the sensor curve COe_Sen_. Minor deviations can only be seen in some cases, such as from *t* = 80 min onwards, where only H_2_ was admixed after the change in atmosphere, or in the case of combined gas dosages such as from *t* = 55 min or 130 min onwards (constant CO level with H_2_ variation in each case). It is important that no major deviations from the theoretical COe line are apparent in the sensor signal, even when different gases were admixed simultaneously (which would mean that a gas is not detected by the sensor). This ensures that the sensor can also measure several components in an exhaust gas and that the sensor signal is then composed of a combination of several partial signals.

### 3.3. Application in a Wood-Burning Stove

**Figure 12:**
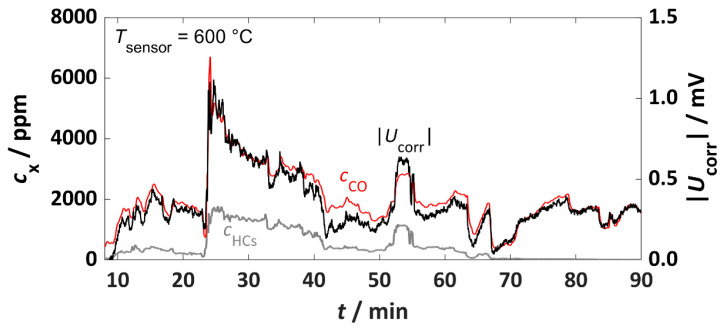
Measurement during the firing of spruce wood in the commercially available wood stove, the corrected sensor signal from the Au/CCO sensor with nine thermocouples in black, concentration of CO and HCs in red and gray, respectively. CO was measured with an NDIR detector, HCs were measured with an FID. The sensor temperature was set to 600 °C.

The final section deals with measurements obtained in the flue gas of the wood-burning stove. The sensor signal was offset-corrected and is shown in Figure 12, together with the raw data from the FID and NDIR detectors, which measure the concentrations of hydrocarbons, CO, and CO_2_, respectively. The Au/CCO sensor was operated at 600 °C, using the integrated back side heater. As can be seen, the measured CO concentrations are in very good agreement with the sensor signal and with its temporal evolution during the entire 90 min during which the furnace was operated.

Some minor deviations occur in a period (*t* = 25 min to *t* = 65 min) when noticeable amounts of HCs are also emitted. Since the FID cannot provide the exact composition of the hydrocarbons, a detailed assessment of their individual contributions to the sensor signal is not possible. Previous studies have shown that methane is typically the dominant hydrocarbon species (approximately 20% to 70%, depending on the setup and fuel) [24,55,56,57]. As methane oxidation on this type of catalyst occurs only at temperatures above 550–600 °C, its contribution to the sensor signal is expected to be negligible under the given conditions. After *t* = 65 min, the burnout phase started, during which predominantly CO and only negligible amounts of hydrocarbons are emitted, in agreement with the literature [55,56]. Here, the sensor signal reflects the CO concentrations excellently.

Overall, the measurement demonstrates that the sensor reliably captures the main trends in the flue gas composition, particularly in regimes dominated by CO. This experiment, therefore, represents a key validation under real flue gas conditions, confirming that the sensing principle developed in this work is transferable from controlled laboratory measurements to practical applications. Further studies could focus on resolving the influence of individual hydrocarbon species in more detail.

## 4. Conclusions

In this work, a thermoelectric gas sensor concept that is intended to measure the sum signal of total hydrocarbons and carbon monoxide (CO equivalent, abbreviated as COe) based on catalytic exothermic reactions (heat release) was further developed by replacing the platinum leg of a thermocouple with a ceramic thermoelectric oxide (Ca_3_Co_4_O_9_, CCO) with a high Seebeck coefficient. The use of the powder aerosol deposition (PAD) method enabled the fabrication of dense, finely patterned CCO films at room temperature, thereby ensuring compatibility with established sensor manufacturing processes.

Systematic investigations of the thermoelectric properties revealed that the Seebeck coefficient of CCO is largely independent of oxygen and water concentrations under relevant operating conditions. This stability represents a key advantage for sensor applications in fluctuating gas atmospheres. Furthermore, short-term thermal cyclability was shown.

Gas sensing experiments demonstrated that replacing conventional Au/Pt thermocouples with Au/CCO significantly increases the sensor signal, resulting in an improvement of nearly one order of magnitude. The sensor exhibited strong linear sensitivities to CO and hydrocarbons, as well as a pronounced response to hydrogen, which can be attributed to its highly exothermic oxidation, while maintaining a low dependency on oxygen and water concentration.

Finally, exemplary measurements under real flue gas conditions confirmed that the sensor reliably tracks the main trends in COe, particularly during CO-dominated combustion phases. These results demonstrate that the proposed sensing principle is transferable from controlled laboratory conditions to practical applications.

Overall, the integration of high Seebeck coefficient oxide materials via the powder aerosol deposition method presents a promising approach for enhancing the performance of thermoelectric gas sensors. Future work should focus on improving selectivity and resolving the contributions of individual gas species under complex operating conditions. As the catalyst has an impact on gas selectivity, the composition could be further optimized for individual applications. Additionally, long-term thermal stability and cyclability should be investigated to ensure proper functionality for practical applications. Catalyst aging could negatively impact the sensor performance; hence additional measurements should be conducted to elucidate aging under various conditions. Finally, the reproducibility of both the manufacturing of the sensors and of the sensors themselves must be examined in the future by fabricating additional sensors and subsequent characterizations. Future measurements could be improved by adding multiple admixing steps to investigate potential signal suppression mechanisms.

## Figures and Tables

**Table 1:** Sensitivities |*m*^a^_x_| calculated from the average values of the seven linear interpolations for both test gases CO and C_3_H_6_ (four oxygen concentration variations and three water concentration variations), including the type of sensor and the number of thermocouples on each sensor.

Sensor Type	Number of Thermocouples	|*m*^a^_CO_| in µV/1000 ppm	|*m*^a^_C3H6_| in µV/1000 ppm
Au/Pt	1	2.8 ± 0.2	11.5 ± 0.6
	3	13.1 ± 0.6	51.1 ± 1.4
	6	25.4 ± 1.3	89.7 ± 4.2
	9	31.6 ± 2.0	121.0 ± 2.9
Au/CCO	1	32.9 ± 2.7	129.2 ± 9.4
	3	93.8 ± 7.5	364.8 ± 21.7
	6	188.8 ± 4.2	756.3 ± 21.9
	9	304.8 ± 7.8	1167.1 ± 34.6

## Data Availability

All relevant data presented in the article are stored according to institutional requirements and, as such, are not available online. However, all data used in this paper can be made available upon reasonable request to the authors.

## Supplementary Materials

The following supporting information can be downloaded at https://www.mdpi.com/article/10.3390/s26154864/s1, Figure S1. Schematic of the power aerosol deposition (PAD) laboratory setup. A carrier gas streams into an aerosol chamber, which is placed on a vibrating table to ease the aerosol generation. The aerosol is transported into the deposition chamber through a nozzle, which is placed below a substrate on a movable substrate table. Inside the deposition chamber, a vacuum is being pulled, which is the main driver for the acceleration of the aerosol into the deposition chamber [58]. Reproduced under terms of the CC-BY license. Copyright 2015 by the authors, published by Journal of Ceramic Science and Technology 6, Göller Verlag GmbH, Baden-Baden, Germany. Figure S2. Schematic of the Seebeck coefficient measurement transducer (SCMT) that was used to measure the Seebeck coefficient of CCO. The heater in front of the transducer (in this case to the right of the transducer) is not shown: (a) during the heating cycles, the voltages across the layer are measured using either the platinum or gold lines. Utilizing the platinum–gold junctions, the temperatures on either side of the transducer can be measured, from which the temperature difference can be calculated. Together with the voltage, the Seebeck coefficient of the layer can be calculated; and (b) during the phase in which the heater is turned off, the resistance of the layer is measured using a four-wire measurement. Figure S3. Seebeck coefficient S measured with the second SCMT. Points indicate the mean value of S during each temperature step; lines connect the points to serve as a guide. Additionally, the deviations from the mean value are shown as error bars for each point. Figure S4. Schematic of the test bench used to measure all sensors. Several mass flow controllers can be used to create an artificial flue gas. Up to four sensors can be mounted into the measurement chamber, however in these experiments, no more than two sensors were measured simultaneously. The Keithley multimeter is connected to a PC to log the data from the sensors and the lambda probe. Figure S5. Laboratory setup of the wood-burning stove used for a measurement with real flue gas. In addition to the sensor, multiple measurement devices, like a flame ionization detector (FID), two non-dispersive infrared detectors (NDIR), and a lambda probe, are installed at different places in the exhaust pipe. The FID measures the total hydrocarbon content and the NDIR detectors measure the concentration of CO and CO_2_, while the lambda probe measures the oxygen concentration. Figure S6. Average voltages |∆*U*_avg_| per C_3_H_6_ concentration step for each measurement cycle at different O_2_ or H_2_O concentrations for the Au/CCO sensor with nine thermocouples. Symbols indicate the average value per step/plateau (green during O_2_ variation, blue during H_2_O variation), the dotted lines represent linear interpolations for the voltages during one measurement cycle, i.e., at a constant O_2_ and H_2_O concentration. Table S1. Temperature profile for firing the gold paste (DuPont 5744R). Table S2. Temperature profile for firing the platinum paste (DuPont 9141R). Table S3. Temperature profile for firing the catalyst paste (1 wt % Pt and 1 wt % Pd in Al_2_O_3_) and the dielectric paste to cover the films (DuPont QM42). Reference [58] is cited in the Supplementary Materials.
