# Supplementary material for: Ca3Co4O9-Based Planar Thermoelectric Gas Sensor with High Sensitivity Fabricated by Powder Aerosol Deposition for Application in Harsh Environments
Source: Sensors (Basel). 2026 Aug 2;26(15):4864. doi: 10.3390/s26154864 (PMC13469921; doi:10.3390/s26154864)
Supplement: Supplementary file 1 [file sensors-26-04864-s001.zip › sensors-4443983-supplementary.pdf]

Supplementary Information

PAD setup

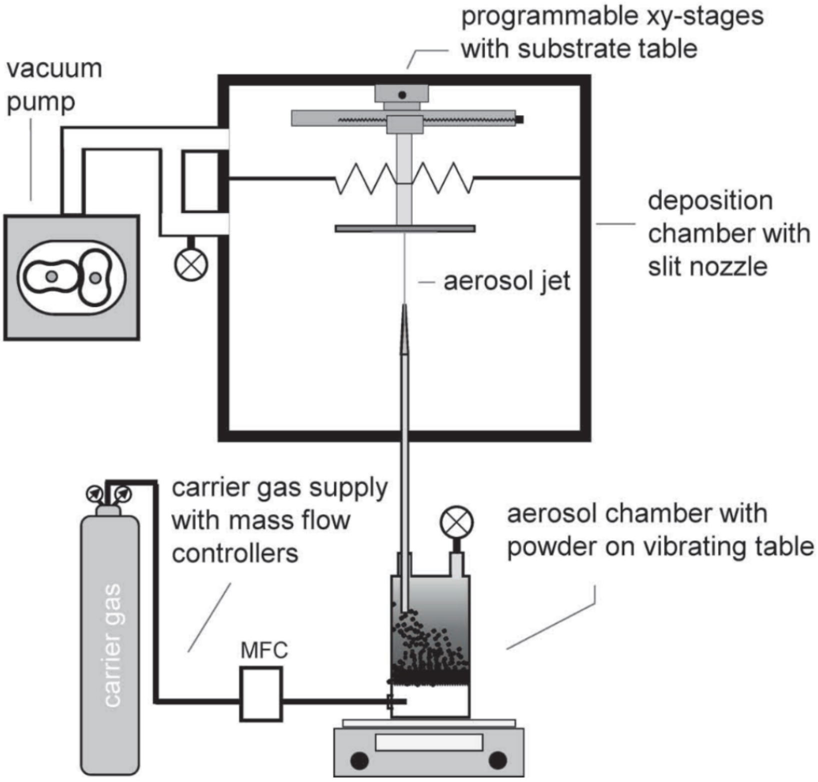

Figure S1. Schematic of the power aerosol deposition (PAD) laboratory setup. A carrier gas streams into an aerosol chamber, which is placed on a vibrating table to ease the aerosol generation. The aerosol is transported into the deposition chamber through a nozzle, which is placed below a substrate on a movable substrate table. Inside the deposition chamber, a vacuum is being pulled, which is the main driver for the acceleration of the aerosol into the deposition chamber [1]. Reproduced under terms of the CC-BY license. Copyright 2015 by the authors, published by Journal of Ceramic Science and Technology **6**, Göller Verlag GmbH, Baden-Baden, Germany.

Seebeck coefficient measurement transducer (SCMT)

The Seebeck coefficient measurement transducer (SCMT) consists of two screen-printed Au/Pt-thermocouples and four platinum conductor lines on a planar aluminum oxide substrate, which are then covered by a layer of the material that is to be examined, i.e., CCO in this study (see Figure S2). A Keithley 2700 multimeter is used to record several thermoelectric voltages, some of which are measured across the film, while others are measured at two points under the film in order to determine the temperature of the film at those points. A separate, stationary heating element in front of the transducer generates a modulated temperature gradient across the transducer (and thus across the film). It is utilized to determine the Seebeck coefficient of the CCO layer. The heating element is operated for a certain period, while the thermoelectric voltages across the layer $U_{th,Pt}$ and $U_{th,Au}$ and the temperatures T_{hot} and T_{cold} are measured. Based on the slope of the thermoelectric voltage as a function of the temperature difference, the relative Seebeck coefficient can be calculated. The absolute Seebeck coefficient (S_{CCO}) is obtained by considering the known Seebeck coefficients of the gold and platinum conductor lines [2]. When the heating element is then switched off for a certain period, the conductivity of the layer can be determined using four-wire measurement (the conductivity plays a minor role for the HC sensors; therefore, it is not further discussed in this study). This entire SCMT setup is placed inside a tube furnace to enable a variable ambient temperature, which makes it possible to determine the Seebeck coefficient at temperatures up to 900 °C. Additionally, the atmosphere inside the furnace can be adjusted using an array of mass flow controllers (MFCs).

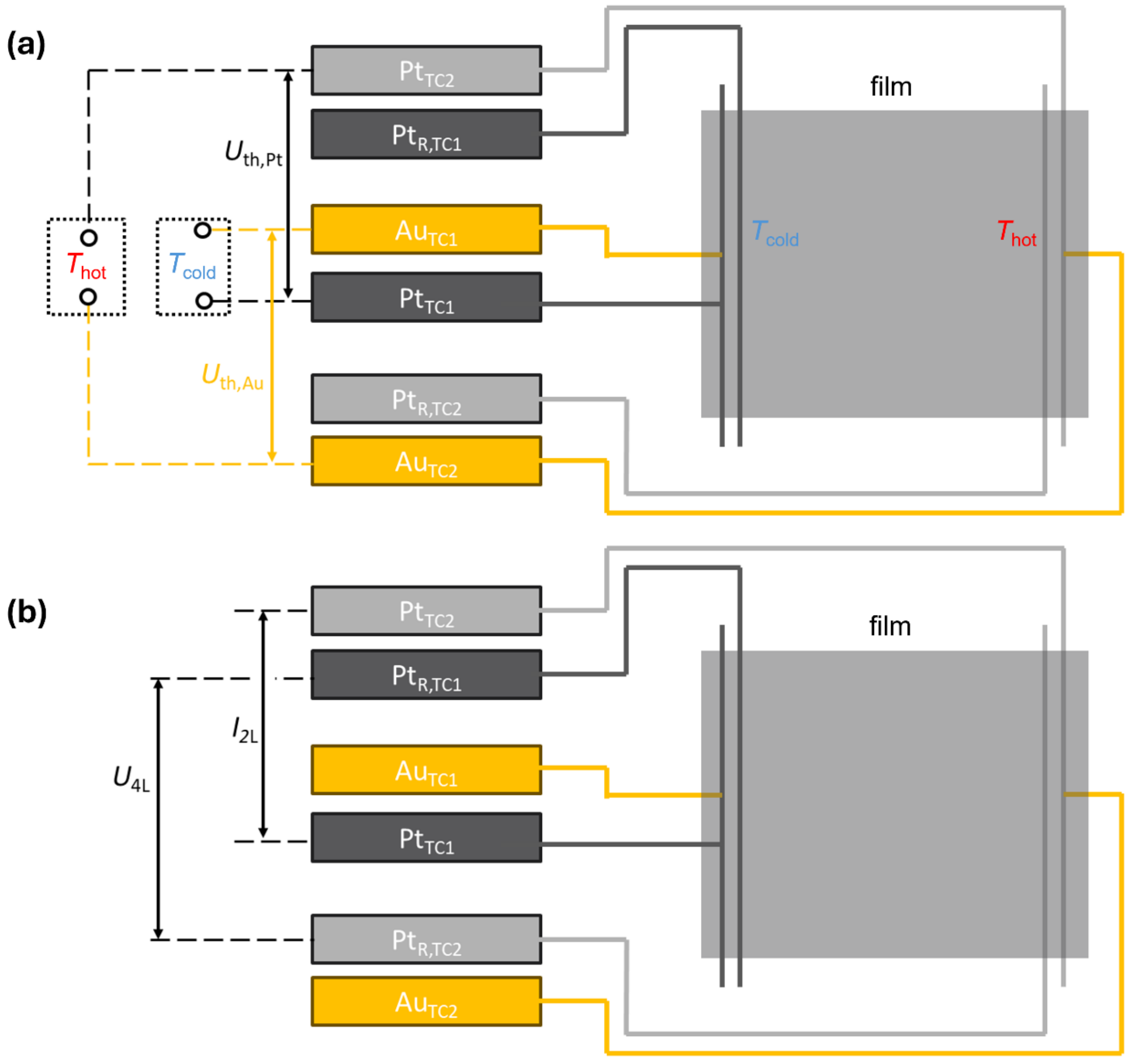

Figure S2. Schematic of the Seebeck coefficient measurement transducer (SCMT) that was used to measure the Seebeck coefficient of CCO. The heater in front of the transducer (in this case to the right of the transducer) is not shown: (a) during the heating cycles, the voltages across the layer are measured using either the platinum or gold lines. Utilizing the platinum–gold junctions, the temperatures on either side of the transducer can be measured, from which the temperature difference can be calculated. Together with the voltage, the Seebeck coefficient of the layer can be calculated; and (b) during the phase in which the heater is turned off, the resistance of the layer is measured using a four-wire measurement.

Seebeck measurement with CCO-Transducer #2

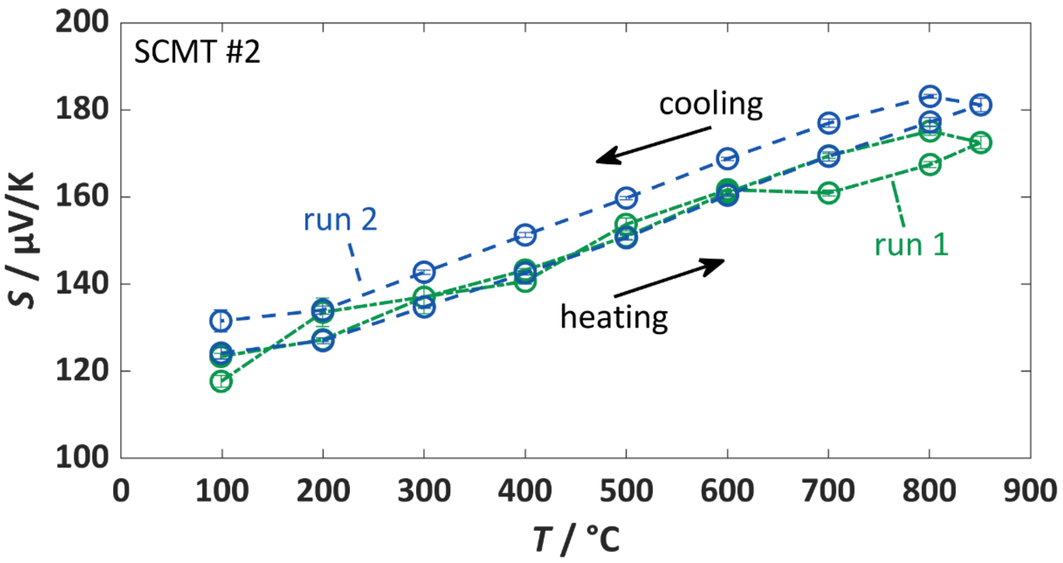

Figure S3. Seebeck coefficient *S* measured with the second SCMT. Points indicate the mean value of *S* during each temperature step; lines connect the points to serve as a guide. Additionally, the deviations from the mean value are shown as error bars for each point.

Laboratory setups

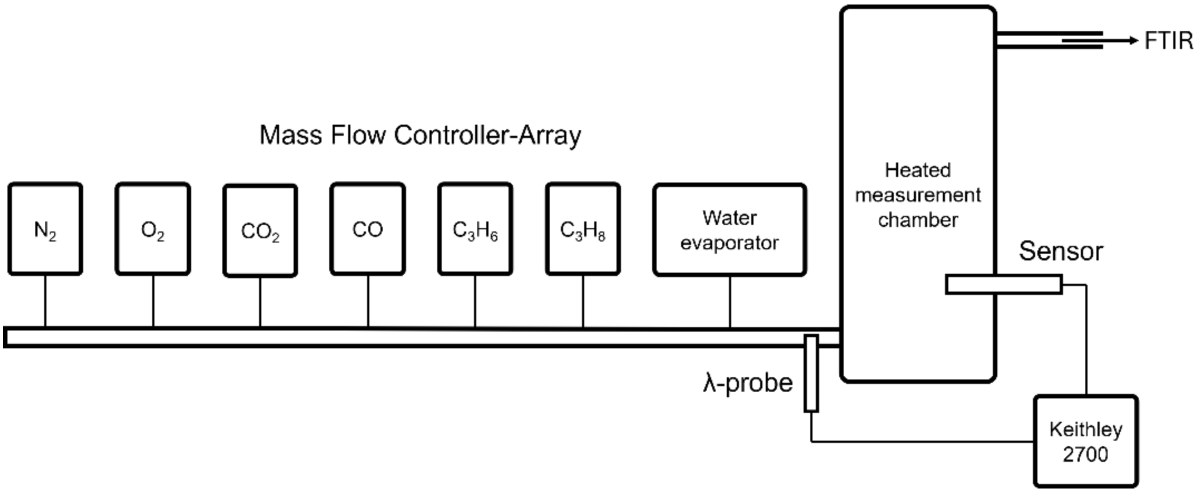

Figure S4. Schematic of the test bench used to measure all sensors. Several mass flow controllers can be used to create an artificial flue gas. Up to four sensors can be mounted into the measurement chamber, however in these experiments, no more than two sensors were measured simultaneously. The Keithley multimeter is connected to a PC to log the data from the sensors and the lambda probe.

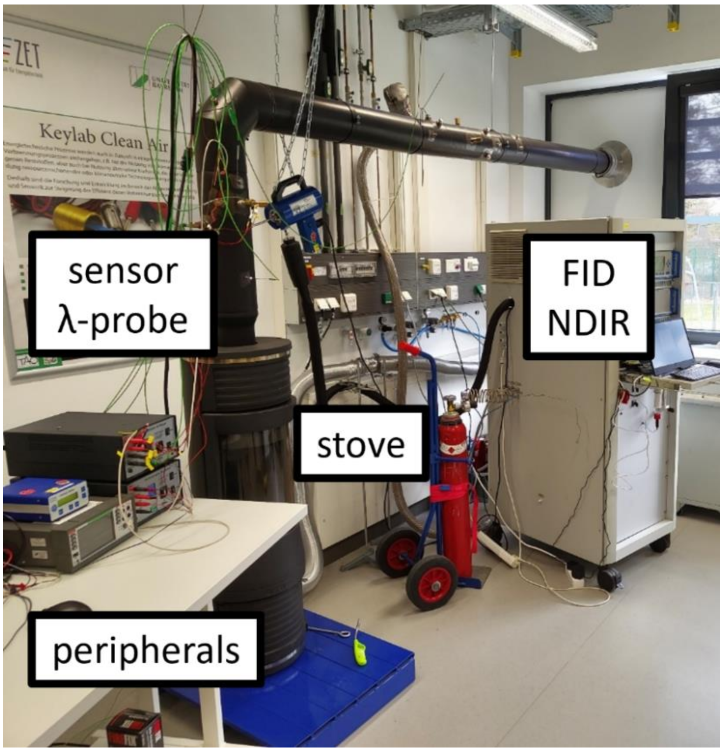

Figure S5. Laboratory setup of the wood-burning stove used for a measurement with real flue gas. In addition to the sensor, multiple measurement devices, like a flame ionization detector (FID), two non-dispersive infrared detectors (NDIR), and a lambda probe, are installed at different places in the exhaust pipe. The FID measures the total hydrocarbon content and the NDIR detectors measure the concentration of CO and CO_2, while the lambda probe measures the oxygen concentration.

Average voltages for C_3H_6 variation

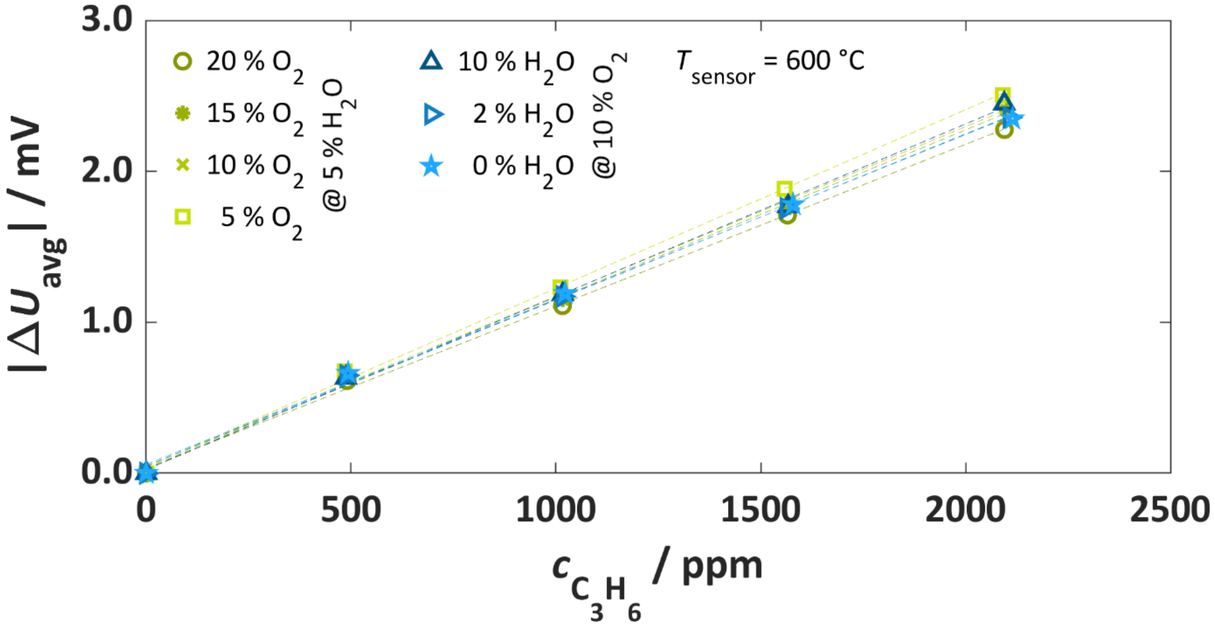

Figure S6. Average voltages $|\Delta U_{avg}|$ per C_3H_6 concentration step for each measurement cycle at different O_2 or H_2O concentrations for the Au/CCO sensor with nine thermocouples. Symbols indicate the average value per step/plateau (green during O_2 variation, blue during H_2O variation), the dotted lines represent linear interpolations for the voltages during one measurement cycle, i.e., at a constant O_2 and H_2O concentration.

Temperature profiles for paste firing

Table S1. Temperature profile for firing the gold paste (DuPont 5744R).

Duration	Temperature start	Temperature end
25 min	room temperature	850 °C
10 min	850 °C	850 °C
25 min	850 °C	room temperature

Table S2. Temperature profile for firing the platinum paste (DuPont 9141R).

Duration	Temperature start	Temperature end
60 min	room temperature	950 °C
20 min	950 °C	950 °C
60 min	950 °C	room temperature

Table S3. Temperature profile for firing the catalyst paste (1 wt % Pt and 1 wt % Pd in Al_2O_3) and the dielectric paste to cover the films (DuPont QM42).

Duration	Temperature start	Temperature end
35 min	room temperature	850 °C
20 min	850 °C	850 °C
35 min	850 °C	room temperature

References

1. Hanft, D.; Exner, J.; Schubert, M.; Stöcker, T.; Fuierer, P.; Moos, R. An Overview of the Aerosol Deposition Method: Process Fundamentals and New Trends in Materials Applications. *J. Ceram. Sci. Tech.* **2015**, *6*, 147–182. https://doi.org/10.4416/JCST2015-00018

2. Bernhard, F. *Handbuch der Technischen Temperaturmessung*; Springer-Vieweg Berlin: Heidelberg, Germany, 2014; pp. 1034–1040. https://doi.org/10.1007/978-3-642-24506-0
